# Cooperation of loss of *NKX3.1* and inflammation in prostate cancer initiation

**DOI:** 10.1242/dmm.035139

**Published:** 2018-11-16

**Authors:** Clémentine Le Magnen, Renu K. Virk, Aditya Dutta, Jaime Yeji Kim, Sukanya Panja, Zoila A. Lopez-Bujanda, Andrea Califano, Charles G. Drake, Antonina Mitrofanova, Cory Abate-Shen

**Affiliations:** 1 Departments of Medicine and Urology, Institute of Cancer Genetics, Herbert Irving Comprehensive Cancer Center, Columbia University Medical Center, New York, NY 10032, USA; 2 Department of Pathology and Cell Biology, Columbia University Medical Center, NY 10032, USA; 3 Department of Medicine, Herbert Irving Comprehensive Cancer Center, Columbia University Medical Center, New York, NY 10032, USA; 4 Department of Health Informatics, Rutgers School of Health Professions, Rutgers, The State University of New Jersey, Newark, NJ 07101, USA; 5 Graduate Program in Pathobiology, Department of Pathology, Johns Hopkins University School of Medicine, Baltimore, MD, USA; 6 Department of Medicine, Columbia Center for Translational Immunology, Herbert Irving Comprehensive Cancer Center, Columbia University Medical Center, New York, NY 10032, USA; 7 Departments of Systems Biology and Biochemistry and Molecular Biophysics, Herbert Irving Comprehensive Cancer Center, Columbia University Medical Center, New York, NY 10032, USA; 8 Department of Systems Biology, Columbia University Medical Center, New York, NY 10032, USA; 9 Departments of Urology, Medicine, Pathology & Cell Biology, and Systems Biology, Herbert Irving Comprehensive Cancer Center, Columbia University Medical Center, New York, NY 10032, USA

**Keywords:** Prostate cancer, Cancer initiation, NKX3.1, Inflammation, Differentiation

## Abstract

Although it is known that inflammation plays a critical role in prostate tumorigenesis, the underlying processes are not well understood. Based on analysis of genetically engineered mouse models combined with correlative analysis of expression profiling data from human prostate tumors, we demonstrate a reciprocal relationship between inflammation and the status of the *NKX3.1* homeobox gene associated with prostate cancer initiation. We find that cancer initiation in aged *Nkx3.1* mutant mice correlates with enrichment of specific immune populations and increased expression of immunoregulatory genes. Furthermore, expression of these immunoregulatory genes is similarly increased in human prostate tumors having low levels of *NKX3.1* expression. We further show that induction of prostatitis in *Nkx3.1* mutant mice accelerates prostate cancer initiation, which is coincident with aberrant cellular plasticity and differentiation. Correspondingly, human prostate tumors having low levels of *NKX3.1* have de-regulated expression of genes associated with these cellular processes. We propose that loss of function of *NKX3.1* accelerates inflammation-driven prostate cancer initiation potentially via aberrant cellular plasticity and impairment of cellular differentiation.

This article has an associated First Person interview with the first author of the paper.

## Abstract (precis)

**Summary:** Chronic inflammation collaborates with loss of function of the prostate-specific tumor-suppressor *NKX3.1* to promote prostate cancer initiation, increase cellular plasticity and impair cellular differentiation.

## INTRODUCTION

Located at the base of the bladder and upstream of the male reproductive system, the prostate provides the first line of defense against foreign agents and pathogens originating from these organs. Consequently, many adult men display signs of inflammation of the prostate, albeit with varying degrees of severity (Sfanos et al., 2017). Such inflammation is often accompanied by infiltration of specific immune cells into the prostate, whose presence has been associated with increased cancer risk and poor prognosis (McArdle et al., 2004; Nonomura et al., 2011, Strasner and Karin, 2015). Indeed, epidemiological and experimental evidence highlight the importance of inflammation for prostate tumorigenesis, and suggest that an inflammatory microenvironment promotes prostate cancer (Sfanos et al., 2014; De Marzo et al., 2007b), while the histological appearance of proliferative inflammatory atrophy (PIA) has been associated with prostate cancer initiation (De Marzo et al., 2007a). Direct evidence that inflammation plays a causal role in prostate cancer includes its association with the promotion of gene fusions that are prevalent in prostate cancer (Mani et al., 2016). In other cancer types, inflammation has been shown to promote carcinogenesis by influencing cellular differentiation, with consequential effects on lineage plasticity (Le Magnen et al., 2018; Coussens and Werb, 2002). Accordingly, in the prostate, inflammation has been associated with impaired differentiation of prostate epithelial cells, including expansion of the pool of progenitor cells (Liu et al., 2016) and aberrant basal-to-luminal differentiation (Kwon et al., 2014), which are accompanied by the promotion of prostate cancer initiation.

A hallmark of prostate cancer initiation is loss of the *NKX3.1* homeobox gene, whose functions have been associated with promotion of lineage plasticity, cellular differentiation and response to inflammation (Abate-Shen et al., 2008). *NKX3.1* is a prostate-specific tumor suppressor located on chromosome 8p, whose loss or reduction represents a key initiating event in prostate cancer (Cancer Genome Atlas Research, 2015; Baca et al., 2013). Germline loss-of-function of *Nkx3.1* in mutant mice results in pre-malignant lesions resembling prostatic intraepithelial neoplasia (PIN), a known precursor of prostate cancer, and shares molecular features conserved with indolent prostate cancer in humans (Bhatia-Gaur et al., 1999; Irshad et al., 2013; Kim et al., 2002a). Moreover, *NKX3.1* is essential for prostatic epithelial specification and proper differentiation, and is required for maintenance of luminal stem cells (Dutta et al., 2016; Wang et al., 2009; Bhatia-Gaur et al., 1999; Talos et al., 2017). Notably, inflammation has been shown to be associated with loss of *NKX3.1* expression in mouse and human prostate (Khalili et al., 2010; Markowski et al., 2008; Bethel et al., 2006).

In the current study, we have investigated the bidirectional relationship between inflammation and *NKX3.1* loss of function, and its relevance for prostate cancer initiation, by comparative analyses of the consequences of its loss of function in mutant mice and its expression status in human prostate cancer. We demonstrate that loss of function of *Nkx3.1* in the mouse prostate is associated with enrichment of specific immune cell populations, and additionally that its loss or reduction in mouse prostate and human prostate tumors is associated with increased expression of immunoregulatory genes. Furthermore, induction of chronic inflammation using a relevant prostatitis model accelerates prostate cancer initiation in *Nkx3.1* mutant mice, which is associated with aberrant cellular plasticity and impaired differentiation. Thus, we propose that loss of function of *NKX3.1* augments inflammation-induced cancer initiation potentially via affecting aberrant cellular plasticity and impaired cellular differentiation.

## RESULTS

### *NKX3.1* loss correlates with an increase in specific immune cells and expression of immunoregulatory genes in prostate

**Fig. 1.**
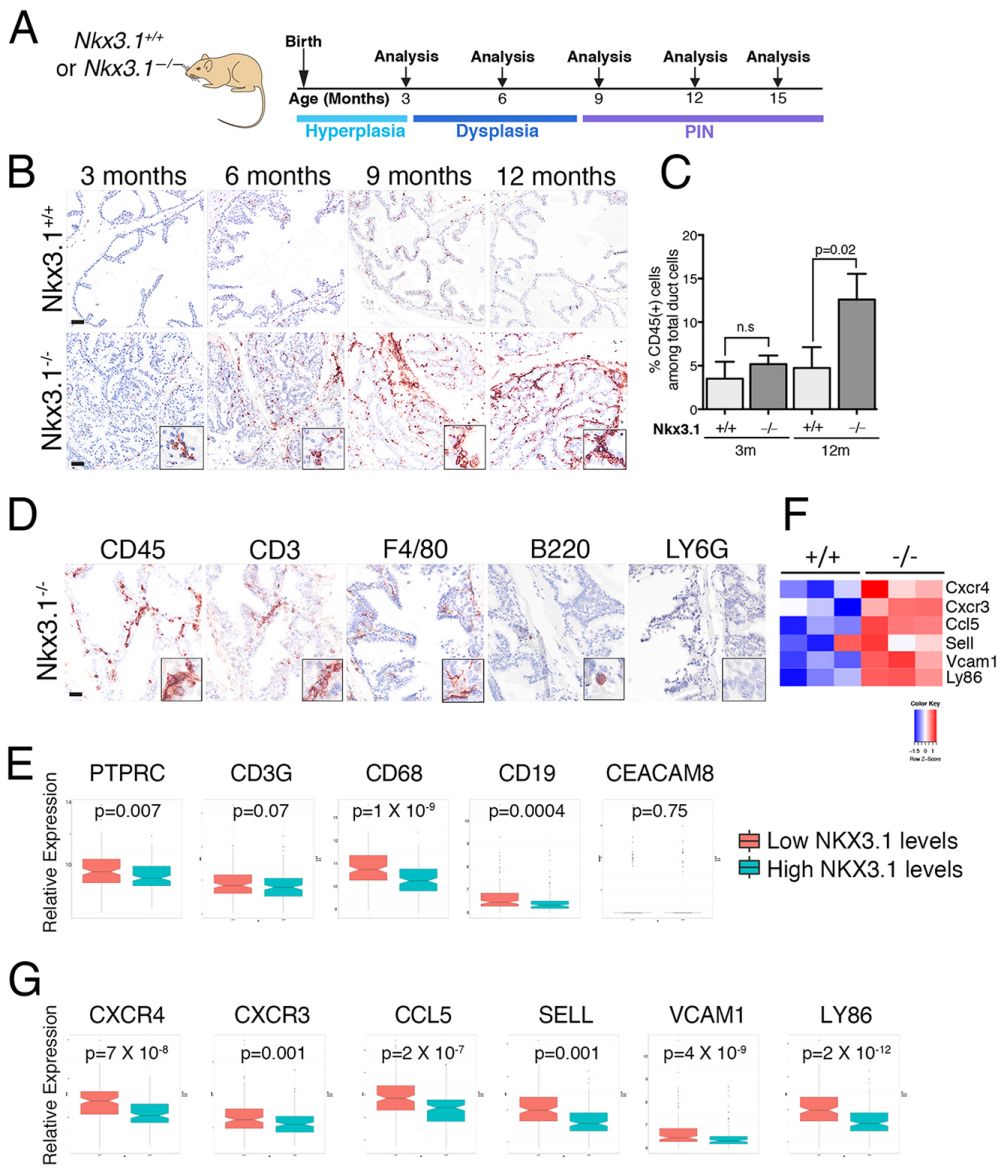
**Loss of *NKX3.1* correlates with** **an increase in specific immune cells and expression of immunoregulatory genes in prostate*.*** (A) Experimental design and timeline of analyses. Shown is the approximate time-range at which the specific phenotypes occur. Note that *Nkx3.1* mutant mice develop prostate intraepithelial neoplasia (PIN) as a consequence of aging. (B) Representative images of immunohistochemical staining for CD45 in the anterior prostates of *Nkx3.1^+/+^* and *Nkx3.1^−/−^* mice at the indicated ages. Scale bars: 50 µm. Insets show higher magnification of a representative region. (C) Quantification of CD45-positive cells infiltrating the prostatic epithelium as assessed by immunohistochemical staining for CD45 expression in *Nkx3.1^+/+^* and *Nkx3.1^−/−^* mice. Analyses were done using a 2-tailed Welch's *t*-test. Data are represented as means and the error bars represent standard deviation (s.d.) for each genotype (*n*=3 mice/group). (D) Representative images of immunohistochemical staining for CD45, CD3, F4/80, B220 and Ly6G in the anterior prostate of *Nkx3.1^−/−^* mice (9 months); additional time points and quantification of cell types is provided in Fig. S1. Scale bar: 25 µm. Insets show higher magnification of a representative region. (F) Heat-map representations of a subset of genes involved in immunoregulatory and inflammatory processes that are differentially expressed in *Nkx3.1^−/−^* versus *Nkx3.1^+/+^* mouse prostate (15 months; *n*=3 mice/group). Differential gene expression was estimated with a 2-sample 2-tailed Welch's *t*-test. Additional pathway and differential gene expression data are provided in Fig. S2. (E,G) Whisker plots showing the relative expression of the indicated human genes in TCGA Gleason 6 and 7 human prostate tumors stratified based on having high or low levels of *NKX3.1* expression. *n*=145 prostate tumors/group. Statistical analyses were performed using a 2-tailed Welch's *t*-test.

To investigate the relationship between loss of function of *Nkx3.1* and inflammation in the mouse prostate *in vivo*, we studied the germline *Nkx3.1* mutant mouse model, which develops PIN as a consequence of aging and cooperates with loss of function of other tumor-suppressor genes in prostate tumorigenesis (Kim et al., 2002a,b; Bhatia-Gaur et al., 1999). In particular, we examined the abundance and nature of immune cells that infiltrate the prostate epithelium (hereafter referred to as ‘prostate-infiltrating immune cells’) in *Nkx3.1* wild-type (*Nkx3.1^+/+^*) and mutant (*Nkx3.1^−/−^*) mice at specific time points, ranging from 3 to 15 months, during which time the prostate phenotype of the *Nkx3.1* mutant mice progresses from hyperplasia to dysplasia to PIN (Fig. 1A) (Bhatia-Gaur et al., 1999). We examined the expression of CD45, a pan-leukocyte marker, and found that the percentage of CD45-positive (CD45+) prostate-infiltrating immune cells was increased in aged *Nkx3.1^−/−^* prostates compared with *Nkx3.1^+/+^* prostates (3-fold increase at 12 months, *P*=0.02; *n*=3 per group; Fig. 1B,C and Table S1), which coincides with the occurrence of PIN in these mice.

To further investigate the phenotype of prostate-infiltrating immune cells, we examined cell-specific markers, namely the T-cell marker CD3, the macrophage marker F4/80, the B-cell-specific marker B220 and the granulocyte marker Ly6G, by immunohistochemistry as well as expression profiling (Fig. 1D; Fig. S1A). We found that the prostate-infiltrating immune cells in the *Nkx3.1^−/−^* prostate were mainly represented by CD3+ T cells and F4/80+ macrophages, which are the main mediators of chronic inflammation (Coussens and Werb, 2002) and in human prostate are present in both normal and inflamed contexts (Sfanos et al., 2017). A rare population of B220+ B cells was also occasionally observed in *Nkx3.1^−/−^* prostates, whereas Ly6G+ neutrophils were not detected (Fig. 1D; Fig. S1A). This was confirmed by quantification of prostate-infiltrating immune populations by flow cytometry, where we found that macrophages and T cells, which include CD4+ and CD8+ cells, were the most prominent populations in *Nkx3.1^−/−^* prostates, whereas B cells and granulocytic myeloid-derived suppressor cells (G-MDSCs), which include neutrophils, were less abundant (Fig. S1B).

To examine the relevance of these findings for human prostate cancer, we analyzed the expression levels of human genes encoding for markers of the corresponding immune cells by querying expression profiles of prostate tumors with low/intermediate Gleason score (i.e. Gleason 6 and 7) from The Cancer Genome Atlas (TCGA; Cancer Genome Atlas Research, 2015). In particular, following from our previous study in which we showed that *NKX3.1* mRNA expression levels provide a read-out of NKX3.1 function in human prostate cancer patients (Dutta et al., 2017) and considering the relevance of *NKX3.1* for early-stage prostate cancer (Abate-Shen et al., 2008), we segregated the low/intermediate Gleason score (Gleason 6 and 7) TCGA samples into two groups based on having ‘low’ (i.e. below the median) or ‘high’ (i.e. above the median) levels of *NKX3.1* mRNA expression (*n*=145 per group). We found that the tumors having ‘low’ *NKX3.1* expression had relatively increased expression of *PTPRC* (coding for CD45), *CD3G*, *CD68* and *CD19,* which mark leukocytes, T cells, macrophages and B cells, respectively, but not *CEACAM8* (*CD66b*) a marker of granulocyte cells, which was generally expressed at very low levels or not detected in the human prostate tumors (Fig. 1E).

Furthermore, analyses of gene expression profiles from aged *Nkx3.1^+/+^* and *Nkx3.1^−/−^* mouse prostates (Ouyang et al., 2005) revealed that 14 of the top 20 most significantly upregulated biological pathways (*P*<0.005) in *Nkx3.1^−/−^* prostates were related to immunoregulatory or inflammatory processes, including ‘chemokine signaling’, ‘signaling in immune system’ and ‘immunoregulatory interactions between a lymphoid and a non-lymphoid cell’ (Fig. S2A; *n*=3 per group; Table S5). Among the positively upregulated leading genes in these pathways were those associated with inflammation-related functions, including the T-cell chemokine receptors *Cxcr4* and *Cxcr3*, the inflammatory chemokine *Ccl5* (RANTES), the adhesion proteins *Sell* and *Vcam1*, and a member of the danger receptor complex, *Ly86* (Fig. 1F; Fig. S2B; *n*=3 per group). Notably, the human homologs of these genes were each significantly upregulated in Gleason 6 and 7 prostate tumors having ‘low’ levels of *NKX3.1* (Fig. 1G). Taken together, these findings in mouse and human prostate show that loss or reduction of *NKX3.1* is associated with enrichment of specific immune populations and increased expression of immunoregulatory genes.

### Chronic inflammation accelerates the phenotypic consequences of *Nkx3.1* loss *in vivo*

**Fig. 2.**
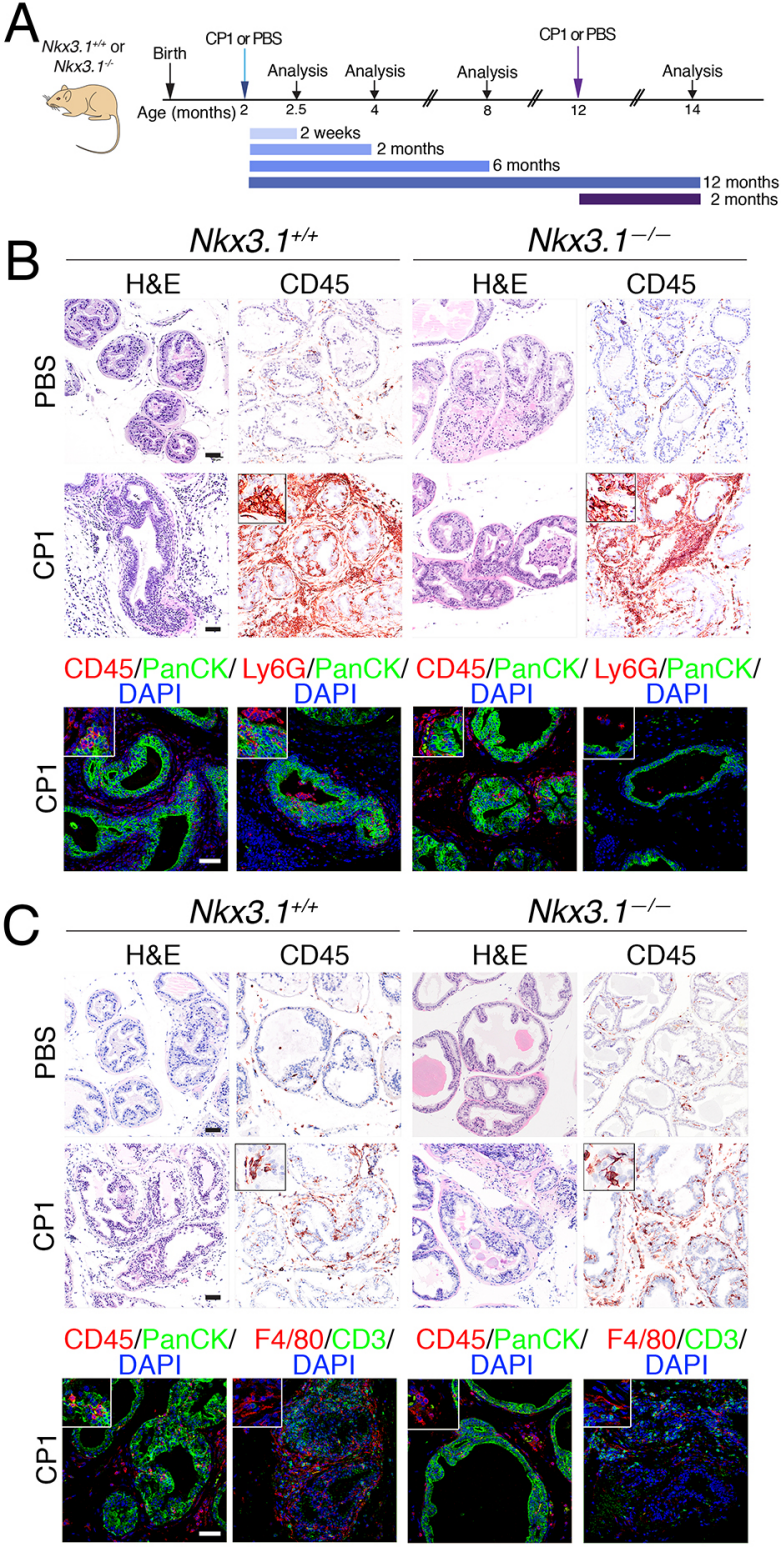
**Modeling prostatitis in *Nkx3.1* mutant mice.** (A) Experimental design for the prostatitis model. *Nkx3.1^+/+^* and *Nkx3.1^−/−^* mice were inoculated with the CP1 bacteria or PBS (as a control) at 2 months of age and analyzed at 2 weeks, 2 months, 6 months and 12 months post-infection. In the ‘aged’ experimental setting (dark purple), mice were inoculated at 12 months of age and analyzed at 2 months post-infection. (B) Representative images of H&E or immunohistochemical and immunofluorescence staining with the indicated antibodies at 2 weeks post-inoculation. Insets show higher magnification of a representative region. (C) Representative images of H&E or immunohistochemical and immunofluorescence staining with the indicated antibodies at 2 months post-inoculation. Insets show higher magnification of a representative region. Scale bars: 50 µm. The total number of mice analyzed in each group is provided in Table S2.

Considering that previous studies have shown that inflammation results in reduced expression of *NKX3.1* in a mouse model of prostatitis (Khalili et al., 2010), we sought to investigate the consequences of inducing inflammation in the context of *Nkx3.1* loss-of-function for prostate cancer initiation. Toward this end, we induced prostatitis in *Nkx3.1* wild-type or mutant mice using CP1 bacteria, which had been isolated from the prostate of a patient with prostatitis and therefore likely to represent a clinically relevant model of prostatic inflammation (Rudick et al., 2011; Simons et al., 2015). Infection of mice with CP1 has been shown to induce a short phase of intense inflammation (i.e. ‘acute’ inflammation), followed by a less intense but persistent inflammation (i.e. ‘chronic’ inflammation), similar to the chronic inflammation that characterizes human prostates (Sfanos et al., 2017). Therefore, we infected adult *Nkx3.1^+/+^* and *Nkx3.1^−/−^* mice with CP1, or PBS as a control, via transurethral inoculation, and investigated the phenotypic consequences from 2 weeks to 1 year post-infection (Fig. 2A; Table S2).

At 2 weeks post-infection, we observed acute inflammation in both the *Nkx3.1^+/+^* and *Nkx3.1^−/−^* prostate, which was evident from the profound histological phenotype and massive infiltration of CD45-stained immune cells in the CP1-infected but not the control mice (Fig. 2B; *n*=4-5 mice per group; Table S2). Inflammation was observed in all three prostatic lobes, namely the anterior prostate (AP), the dorsolateral prostate (DLP) and the ventral prostate (VP), although to different degrees (Fig. S3A). At this early time point, the inflammatory response was still mainly acute as evident by the prominent presence of Ly6G+ neutrophils (Fig. 2B), which are primary mediators of acute inflammation (Kolaczkowska and Kubes, 2013).

At later time points post-infection, the *Nkx3.1^+/+^* and *Nkx3.1^−/−^* prostates displayed evidence of chronic inflammation (Fig. 2C; Fig. S3B). In particular, at 2 months post-infection, both *Nkx3.1^+/+^* and *Nkx3.1^−/−^* mice had sustained presence of immune cells in the CP1-infected but not the control prostates, evident by immunostaining for CD45 (Fig. 2C; *n*=5-7 mice per group; Table S2). As expected, the immune infiltration at this later time point was less prominent than at 2 weeks and characterized by the presence of other immune subsets, including CD3+ T cells and F4/80+ macrophages (Fig. 2C). This inflammatory reaction persisted over time since sustained increased immune infiltration continued to be observed at 6- and 12-months post-infection (Fig. S3B; *n*=4-5 mice per group; Table S2).

**Fig. 3.**
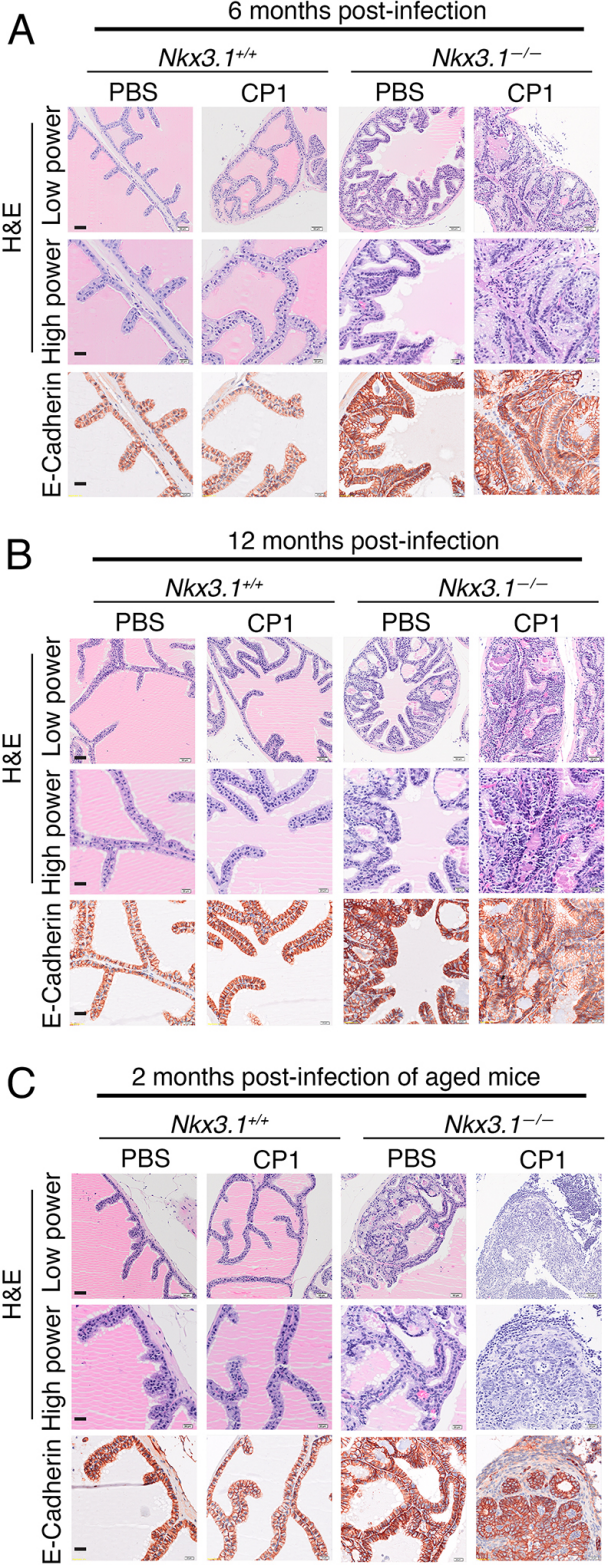
**Chronic inflammation accelerates prostate cancer initiation in *Nkx3.1* mutant prostate.** Representative images of *Nkx3.1^+/+^* and *Nkx3.1^−/−^* anterior prostate showing H&E or immunohistochemical staining at the indicated time points following infection with CP1 (as in Fig. 2). Scale bars: 50 µm in low-power images and 20 µm in high-power images. Additional analyses of the phenotype are provided in Figs S3 and S4. The total number of mice analyzed in each group is provided in Table S2, and a summary of the histological phenotype is provided in Table S3.

Although both the *Nkx3.1^+/+^* and *Nkx3.1^−/−^* prostates displayed evidence of chronic inflammation, the latter were prone to acceleration of the PIN phenotype. This was evident by histological inspection by the increased cellularity, more pronounced dysplasia, and areas of microinvasion in the *Nkx3.1^−/−^* but not *Nkx3.1^+/+^* prostate (Fig. 3; *n*=4-5 mice/group; Table S3). Notably, this phenomenon was not correlated with significant increased proliferation, as suggested by comparable levels of Ki67 expression in CP1- and PBS-infected *Nkx3.1^−/−^* prostates (Fig. S3C). In some cases, the phenotype was accompanied by some areas with disrupted alpha-smooth muscle actin (SMA) expression and focal positivity for the cell-death marker activated-Caspase 3 (Fig. S4). Although the tendency toward more aggressive PIN phenotypes was evident as early as 2 months after induction of prostatitis, the most striking examples were observed at 6 months and 12 months post-infection (Fig. 3A,B; Fig. S4; Table S3), highlighting the importance of aging for the pre-cancerous phenotype of the *Nkx3.1* mutant mice, as we have observed previously (Bhatia-Gaur et al., 1999; Irshad et al., 2013). To further investigate the relationship of aging for consequences of inflammation, we induced prostatitis in ‘aged’ (i.e. at 12 months) *Nkx3.1^+/+^* and *Nkx3.1^−/−^* mice and analyzed the prostate phenotype 2 months after infection (i.e. at 14 months). We found that the aged *Nkx3.1^−/−^* mice displayed a similar accelerated PIN phenotype specifically in the CP1-infected mice (Fig. 3C; Table S3). Taken together, these findings suggest that loss of function of *Nkx3.1* promotes inflammation-mediated acceleration of prostate cancer initiation *in vivo*.

### Inflammation promotes basal-to-luminal differentiation in *Nkx3.1* mutant mice

**Fig. 4.**
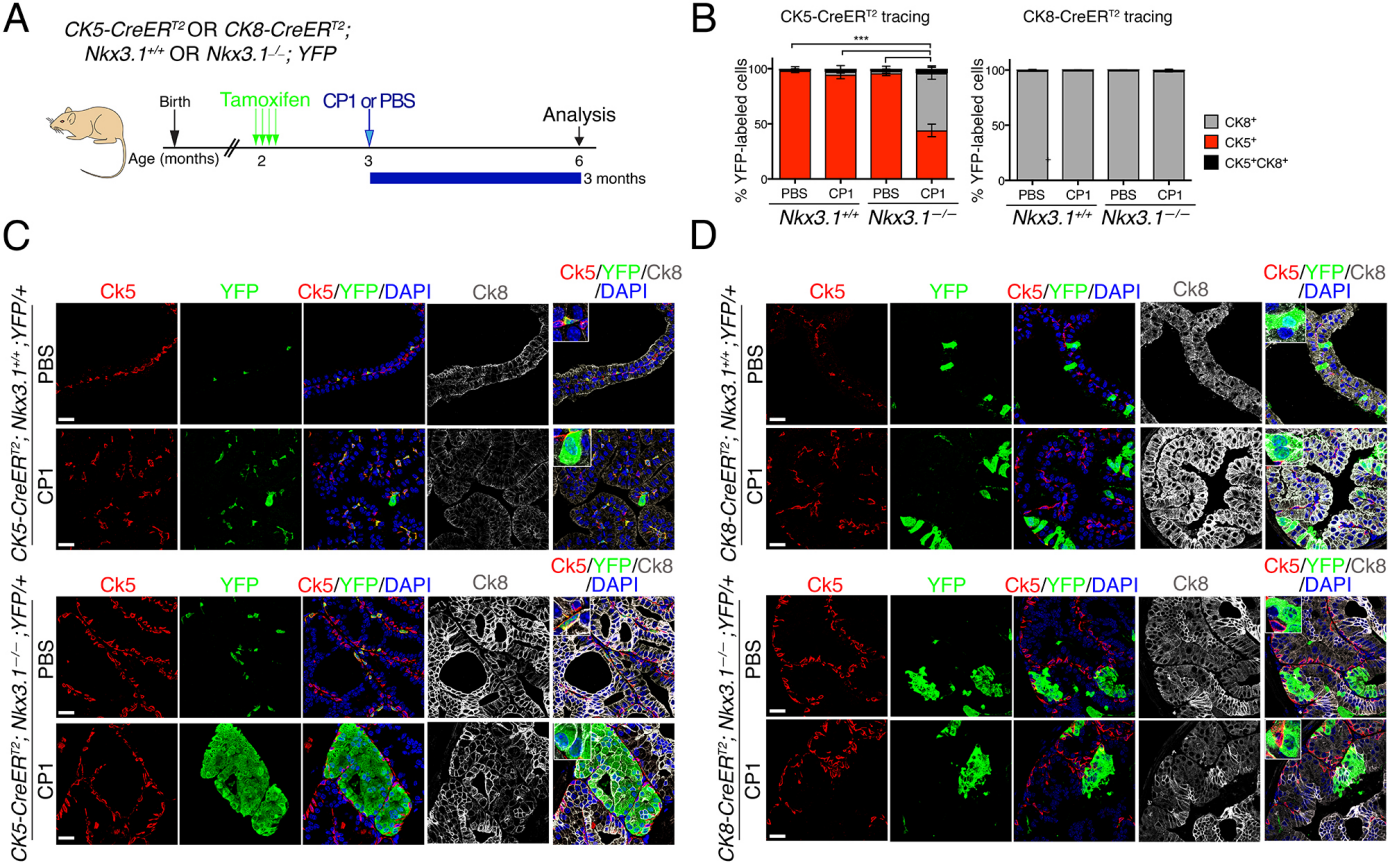
**Inflammation is associated with increased basal-cell plasticity in the *Nkx3.1* mutant prostate.** (A) Experimental design and timeline. *CK5-CreER^T2^* or *CK8-CreER^T2^; Nkx3.1^+/+^* or *Nkx3.1^−/−^; YFP* mice were induced with tamoxifen at 2 months of age and inoculated with a single dose of CP1 (or PBS as a control) 1 month after tamoxifen induction. Expression of specific markers was analyzed and quantified 3 months post-inoculation. Whole-mount images are provided in Fig. S5. (B) Quantification of YFP-positive cells that co-express CK5 (red), CK8 (gray) or both CK5 and CK8 (black) using the *CK5-CreER^T2^* (left) or the *CK8-CreER^T2^* (right) driver. For each group, at least three animals were analyzed (*n* numbers in Table S2), and the quantification of the lineage-marked cells is provided in Table 1. A two-tailed Welch's *t*-test was performed to compare the percentage of YFP-positive cells that co-express Ck5 or Ck8 in all groups. Significant differences are indicated (***P<0.001). Data are represented as means and the error bars represent standard error (s.e.m.) for each group. (C,D) Lineage tracing with *CK5-CreER^T2^* (C) and *CK8-CreER^T2^* (D). Representative images of immunofluorescence staining with the indicated antibodies. Insets show higher magnification of a representative region. Scale bars: 25 µm.

Previous studies have shown that acute prostatic inflammation is associated with basal-to-luminal differentiation *in vivo* (Kwon et al., 2014). Therefore, we asked whether chronic inflammation induced by CP1 promotes changes in the differentiation potential of prostatic epithelial cells consistent with prostatic epithelial plasticity and, if so, whether this is influenced by the status of *Nkx3.1*. Toward this end, we performed lineage tracing using a tamoxifen-inducible Cre (*CreER^T2^*) under the control of cytokeratin 5 (*CK5-CreER^T2^*) or cytokeratin 8 (*CK8-CreER^T2^*) promoters to target gene recombination specifically to basal or luminal cells, respectively (Indra et al., 1999; Van Keymeulen et al., 2011). Notably, the *CK5-CreER^T2^* allele is distinct from the *CK14-CreER^T2^* allele used in a previous study (Kwon et al., 2014). These Cre drivers were crossed with a conditionally-activatable fluorescent reporter allele (*R26^CAG-EYFP^*; Madisen et al., 2010) as well as with the *Nkx3.1* wild-type or mutant mice to generate mice having the following genotypes: *CK5-CreER^T2^* or *CK8-CreER^T2^; Nkx3.1^+/+^* or *Nkx3.1^−/−^; R26^CAG-EYFP/+^* (Wang et al., 2013). Accordingly, tamoxifen induction resulted in lineage tracing specifically of basal (*Ck5*-expressing) or luminal (*Ck8*-expressing) cells, respectively, in the context of wild-type or null *Nkx3.1*. One month after tamoxifen induction, mice were infected with CP1 (or PBS as a control) and analyzed at 2-3 months post-infection (*n*=3 or 4 mice/group; Fig. 4A; Table S2B), during the phase of chronic inflammation (as above). We quantified the relative percentage of lineage-marked basal or luminal cells based on co-staining for YFP and Ck5 and/or Ck8, respectively, in prostates of mice infected with CP1 or the control (PBS) (Table 1).

**Table 1.**
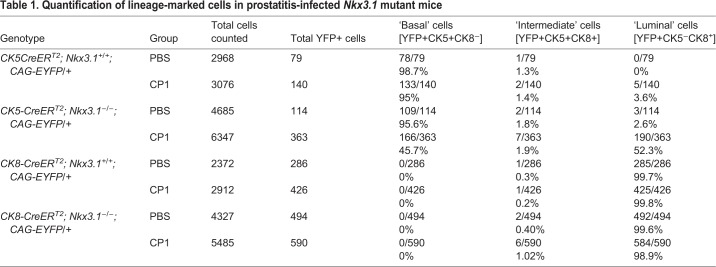
**Quantification of lineage-marked cells in prostatitis-infected *Nkx3.1* mutant mice**

Consistent with previous studies (Wang et al., 2013; Choi et al., 2012), lineage tracing of control (i.e. PBS-treated) *Nkx3.1^+/+^* prostates with *CK5-CreER^T2^* marked predominantly (>98%) Ck5-expressing basal cells, a small percentage (<2%) of cells co-expressing Ck5 and Ck8 (presumptive intermediate cells; Wang et al., 2013) and no Ck8-expressing luminal cells (*n*=3; Fig. 4B,C; Table 1). Conversely, lineage tracing with *CK8-CreER^T2^* marked primarily luminal cells (>99%), very few intermediate cells (<1%) and no Ck5-expressing basal cells (*n*=3; Fig. 4B,D; Table 1). Although these lineage relationships were largely maintained in CP1-infected *Nkx3.1^+/+^* prostates, lineage tracing with *CK5-CreER^T2^* revealed a small percentage (3.6%) of Ck8-expressing cells that had a luminal-like shape, while the converse was not observed for lineage-tracing with *CK8-CreER^T2^* (*n*=4 or 3, respectively; Fig. 4B-D; Table 1).

In contrast to these findings for wild-type prostate, chronic inflammation was associated with profound epithelial plasticity in *Nkx3.1^−/−^* prostate. In particular, *CK5-CreER^T2^* lineage tracing of control-treated *Nkx3.1^−/−^* prostates primarily marked Ck5-expressing basal cells (95.6%, *n*=3) and some intermediate cells (2.6%); although, unlike the *Nkx3.1^+/+^* prostate, a small percentage of Ck5 lineage marked cells (1.8%) were Ck8-expressing luminal cells (*n*=3; Fig. 4B,C; Fig. S5; Table 1). In striking contrast, in the *CK5-CreER^T2^* lineage-traced CP1-infected *Nkx3.1^−/−^* prostates, more than half of the cells (52.3%, *n*=4) were Ck8-expressing luminal cells, representing a 29-fold increase compared to the PBS-treated *Nkx3.1^−/−^* prostates (*P*<0.001, *t*-test; *n*=4; Fig. 4B,C; Fig. S5; Table 1).

Such inflammation-enhanced plasticity was not observed for luminal cells since lineage tracing of *CK8-CreER^T2^* mice predominantly marked Ck8-expressing luminal cells (>98%) in both the PBS- and CP1-treated *Nkx3.1^−/−^* prostates (*n*=3 or 4, respectively; Fig. 4B,D; Table 1). Taken together, these findings suggest that inflammation greatly augments an inherent potential for basal-to-luminal differentiation in the *Nkx3.1* mutant prostate.

### Nkx3.1-driven gain of basal features and loss of luminal features is enhanced upon inflammation

**Fig. 5.**
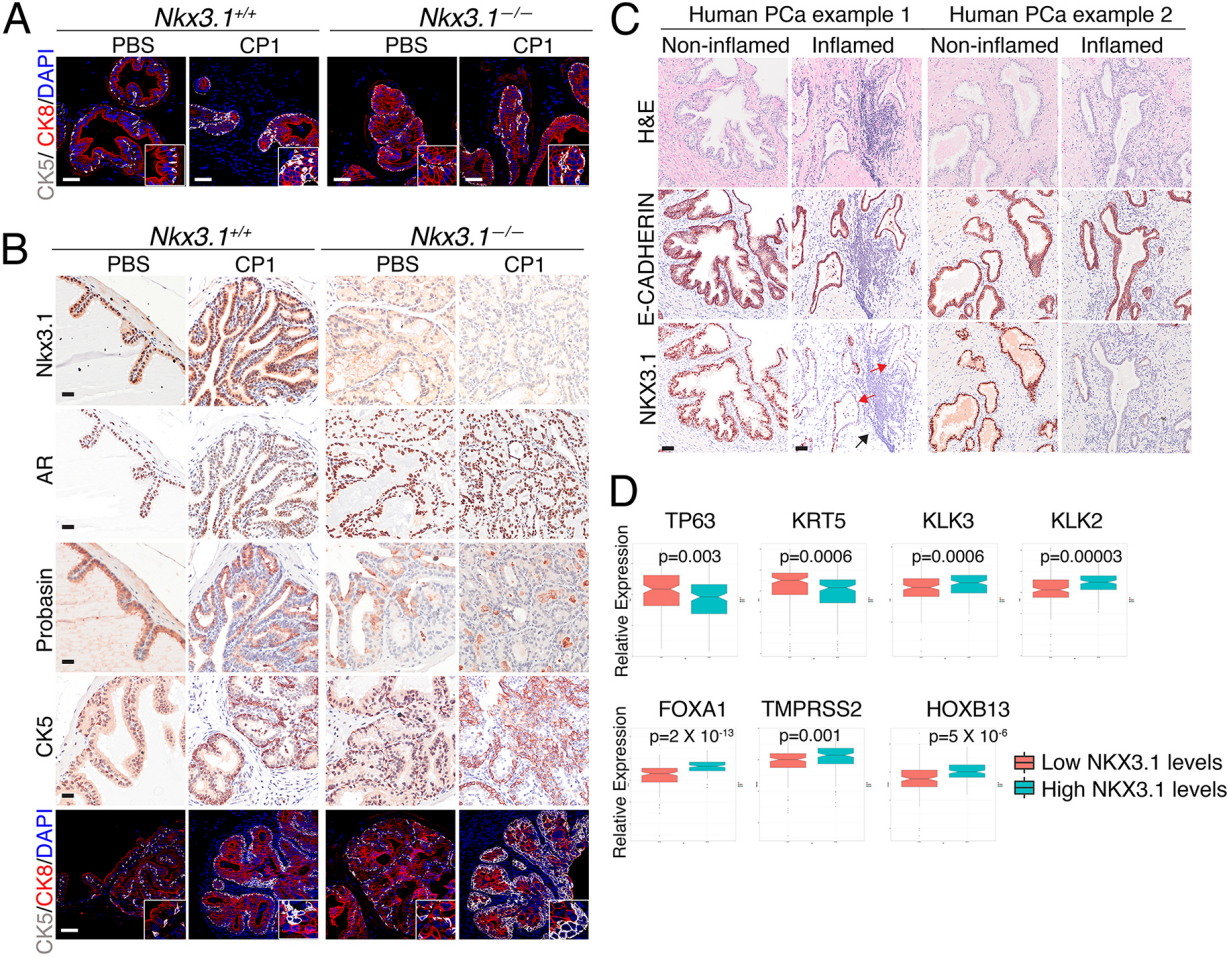
**Inflammation promotes loss of luminal features in *Nkx3.1* mutant prostate.** (A) Representative images of immunohistochemical and immunofluorescence staining with the indicated antibodies at 2 weeks post-inoculation. Insets show higher magnification of a representative region. Scale bars: 50 µm. (B) Representative images of staining with the indicated antibodies at 2 months post-infection in the aged mice (see Fig. 2). Insets show higher magnification of a representative region. Scale bars: 25 µm for immunohistochemical analyses and 50 µm for immunofluorescence analyses. (C) Representative images of immunohistochemical staining of human prostate cancer (PCa) samples with the indicated antibodies. Scale bars: 50 µm. Black arrow shows inflammation; red arrows show loss of NKX3.1 in proximity to inflamed areas. Additional examples are shown in Fig. S7. (D) Whisker plots showing the relative expression of the indicated human genes in TCGA Gleason 6 and 7 human prostate tumors stratified based on having high or low levels of *NKX3.1* expression. *n*=145 prostate tumors/group. Statistical analyses were performed using 2-tailed Welch's *t*-test.

Since *NKX3.1* is required for proper prostatic epithelial differentiation and specification of luminal prostatic epithelial cells (Dutta et al., 2016; Bhatia-Gaur et al., 1999; Talos et al., 2017), we considered whether the enhanced plasticity of basal cells in the chronically inflamed *Nkx3.1^−/−^* prostate was due to impaired differentiation. In particular, we examined markers of luminal or basal cell differentiation by immunohistochemistry in CP1-infected (or control) *Nkx3.1^+/+^* and *Nkx3.1^−/−^* prostate during acute or chronic inflammation (Fig. 5A,B). In the acute-inflammation stage (i.e. 2 weeks post-infection), both *Nkx3.1^+/+^* and *Nkx3.1^−/−^* prostates exhibited features of altered differentiation as evident by an increased number of Ck5-expressing cells (Fig. 5A). Furthermore, this altered differentiation was maintained in the chronic-inflammation stage in *Nkx3.1^−/−^* prostates. In particular, the aged cohort (i.e. infected at 12 months and analyzed at 14 months) displayed reduced expression of cells expressing luminal markers (Nkx3.1, AR, probasin) and increased prevalence of Ck5-expressing cells (Fig. 5B; Fig. S6).

Notably, at the acute-inflammation stage, *Nkx3.1^+/+^* prostates exhibited a loss of Nkx3.1 protein expression (Fig. S3D), consistent with a previous study (Khalili et al., 2010). This loss of Nkx3.1 protein expression occurred specifically in the inflamed prostatic ducts, which were highly proliferative as evident by Ki67 staining, while non-inflamed ducts had normal levels of Nkx3.1 protein and normal proliferation (Fig. S3D). To determine whether this relationship between NKX3.1 loss and inflammation occurs in human prostate cancer, we performed immunohistochemistry on human prostatectomy samples (*n*=11) to detect expression of NKX3.1 or E-cadherin, an epithelial cell marker. Strikingly, in these human prostate cancers, NKX3.1 protein expression was reduced specifically in areas of inflammation (Fig. 5C; Fig. S7).

Considering these findings, we sought to investigate whether *NKX3.1* loss is associated with impaired differentiation in human prostate cancer. In particular, we analyzed the expression levels of genes associated with luminal or basal differentiation in low/intermediate Gleason TCGA primary tumors (Cancer Genome Atlas Research, 2015) segregated by ‘low’ or ‘high’ levels of *NKX3.1* expression, as described above. Notably, genes associated with luminal differentiation (i.e. *FOXA1*, *TMPRSS2*, *HOXB13*, *KLK3*, *KLK2*) had significantly reduced expression in the ‘low’ *NKX3.1* prostate tumors (Fig. 5D). In contrast, expression of basal markers (*TP63*, *KRT5*) was significantly increased in the ‘low’ *NKX3.1* prostate tumors (Fig. 5D). Furthermore, correlation of the expression levels of *NKX3.1* with both immunoregulatory and differentiation-related genes in the TCGA cohort revealed a significant positive correlation between *NKX3.1* and genes encoding luminal markers, and a significant negative correlation with genes encoding both basal markers and specific immune- and inflammation-related markers, as evident by Pearson correlation and heat-map analyses (Fig. S8). Together, these findings establish a link between loss of *NKX3.1*, inflammation and aberrant differentiation in both mouse and human prostate cancer.

## DISCUSSION

Chronic inflammation has long been linked to prostate cancer, although the underlying cellular processes are not well understood. In the current study, we demonstrate that loss of a key tumor-suppressor gene associated with prostate cancer initiation, namely *NKX3.1*, exacerbates inflammation-induced prostate cancer initiation, which is coincident with enhanced epithelial plasticity and defects in cellular differentiation. In particular, we have modeled prostatitis *in vivo* using the CP1 bacterial strain (Simons et al., 2015), which induces long-term chronic inflammation similar to that observed in aged men (Sfanos and De Marzo, 2012). This enabled us to study the consequences of chronic inflammation for both cancer-related and differentiation-related phenotypes in the context of *Nkx3.1* loss-of-function *in vivo*. Importantly, we observed a striking correlation between the status of *NKX3.1* and response to inflammation and cellular differentiation, which is exacerbated upon aging. Furthermore, by complementing analyses of this mouse model with correlative analyses of expression profiling data from human patients, we have demonstrated the relevance of our findings in mice for human prostate cancer. We propose that such an inverse relationship between *NKX3.1* status and response to inflammation has consequences for maintaining the differentiation status of the prostatic epithelium, which impacts prostate cancer initiation particularly in the context of aging.

While previous studies have shown reduced expression of *NKX3.1* following inflammation in mouse and human prostate (Khalili et al., 2010; Markowski et al., 2008; Bethel et al., 2006), our current studies establish that this relationship is bidirectional and link these findings to cancer initiation as well as to altered cellular differentiation. In particular, we find that loss of *NKX3.1* is associated with enrichment of specific immune populations and immune/inflammation markers, and alteration of differentiation markers in both mouse and human prostate. Conversely, in contexts of *Nkx3.1* deficiency as occurs in tissues in which chromosomal region 8p21 is lost, chronic inflammation accelerates prostate cancer initiation, which is coincident with impaired cellular differentiation and aberrant cellular plasticity. Notably, *NKX3.1* is known to control prostate specification (Dutta et al., 2016) and to be required for normal functioning of luminal stem cells (Wang et al., 2009). Therefore, our current findings suggest that impairment of these functions in the context of *NKX3.1* loss impedes the response of the prostate to inflammatory assaults, thereby making it more susceptible to cancer initiation. Moreover, our data showing the increased expression of immunoregulatory genes in *NKX3.1-*deficient contexts in mouse and human prostate warrants further investigation of the roles of specific immune-cell populations in these processes.

Our study also suggests an important link between *NKX3.1* status and aging, as we have observed previously (Bhatia-Gaur et al., 1999; Irshad et al., 2013), since the phenotypic changes associated with inflammation are more profound in aged mice. Considering that aging and inflammation are two well-known etiological factors of cancer (Ahmad et al., 2009), it is not surprising that they may synergize in promoting cancer initiation. In particular, it is widely accepted that aging is associated with important compromises of the immune system and in inflammatory changes, which may affect responses to inflammatory and oncogenic stimuli (Ahmad et al., 2009; Licastro et al., 2005). In addition, experimental evidence suggests that tissue-specific reserves of stem cells decline and their normal activity (e.g. differentiation and tissue repair) is impaired with advancing age (Rossi et al., 2008). Thus, aging, inflammation and impaired differentiation are biological processes that are all linked to cancer initiation and progression, and *NKX3.1* may serve as an important mediator for regulating these processes.

Curiously, *NKX3.1* is primarily expressed in luminal cells, whereas enhanced plasticity following inflammation mainly occurs in basal cells in *NKX3.1-*deficient contexts. In particular, chronic inflammation promotes basal-to-luminal differentiation in *Nkx3.1* mutant mice, a phenomenon that rarely occurs in normal conditions (Lee and Shen, 2015). However, other conditions that perturb the prostate epithelium, such as acute inflammation or anoikis, have been shown to result in increased basal-to-luminal differentiation (Toivanen et al., 2016; Kwon et al., 2014). Interestingly, in the current study, in which we are investigating a chronic inflammatory context, we find that lineage plasticity is rarely seen in wild-type basal cells, although a previous study showed it to be more frequent in an acute inflammatory context (Kwon et al., 2014). This difference may be attributed to the specific features of the CP1 model or to chronic versus acute inflammation, which may impact the cellular phenotype or differentiation status. Notably, markers of chronic inflammation, such as CD3 receptors or CD68, are strongly correlated with *NKX3.1* status in human prostate tumors, which is not the case for markers of acute inflammation such as CEACAM8 or CD177. The current study suggests that, following chronic inflammatory assault, *NKX3.1-*deficient luminal cells may ultimately be replaced by basal cells, which may have important implications for prostate cancer initiation.

In summary, our study highlights the importance of *NKX3.1* status for cellular plasticity, inflammation and aging during prostate cancer initiation. The implication of our findings is that analysis of the status of *NKX3.1* combined with markers of inflammation and differentiation may provide insight regarding prognosis of men during initial stages of prostate cancer. Indeed, given that *NKX3.1* maps to a key region of the chromosome, 8p21, that undergoes allelic loss in up to 80% of early-stage prostate cancer cases, incorporating analyses of inflammation status combined with *NKX3.1* status may enhance models for risk assessment of prostate cancer in a precision cancer-prevention context.

## MATERIALS AND METHODS

### Description of mouse models

All experiments using animals were performed according to protocols approved by the Institutional Animal Care and Use Committee (IACUC) at Columbia University Medical Center, NY, USA. The *Nkx3.1* germline mutant mice have been described previously (Bhatia-Gaur et al., 1999); the studies performed herein were done using *Nkx3.1* wild-type mice (*Nkx3.1^+/+^*) and homozygous-null mice (*Nkx3.1^−/−^*). These mice have been maintained in our laboratory on a predominantly C57BL/6 background. For lineage-tracing studies, the following mouse alleles were obtained from the Jackson Laboratory (https://www.jax.org) and maintained in a mixed C57BL/6/129S strain background: *R26^CAG-EYFP^* (stock number 007903; Madisen et al., 2010), *CK5-CreER^T2^* (stock number 018394/*K5-Cre-ER^T2^*; Indra et al., 1999) and *CK8-CreER^T2^* (stock number 017947/K8-CreER; Van Keymeulen et al., 2011). These *Cre* drivers were crossed for several generations with the reporter allele (*R26^CAG-EYFP^*) as well as with the *Nkx3.1^+/+^* and *Nkx3.1^−/−^* alleles to generate a series of mice with the following genotypes: *CK5-CreER^T2^* or *CK8-CreER^T2^; Nkx3.1^+/+^* or *Nkx3.1^−/−^; R26^CAG-EYFP/+^.* To induce gene recombination for lineage-tracing studies, mice were administered tamoxifen (Sigma; 9 mg/40 g body weight) suspended in corn oil by oral gavage once daily for 4 consecutive days as reported previously (Wang et al., 2009, 2013). All studies were done using littermates that were genotyped prior to enrollment; only male mice were used because of the focus on prostate.

### Molecular and phenotypic analyses of *Nkx3.1* wild-type and mutant mice

Expression profiling of anterior prostate from 15-month-old *Nkx3.1^+/+^* and *Nkx3.1^−/−^* mutant mice on an Affymetrix platform (Mu74AV2) has been described (Ouyang et al., 2005; Irshad et al., 2013). Analyses of differentially expressed genes and biological pathways were reported (Dutta et al., 2016). Briefly, a differential gene expression signature was defined between *Nkx3.1^−/−^* and *Nkx3.1^+/+^* using a 2-sample 2-tailed Welch’s *t*-test. This signature was subjected to pathway enrichment analysis using the C2 database, which includes pathways from REACTOME (Fabregat et al., 2016), KEGG (Ogata et al., 1999) and BioCarta (https://cgap.nci.nih.gov/Pathways/BioCarta_Pathways). Pathway analysis was performed using gene set enrichment analysis (GSEA) (Subramanian et al., 2005), with the significance of enrichment estimated using 1000 gene permutations.

For phenotypic analysis, mice were sacrificed at the indicated time points, and prostate tissues were collected and fixed in 10% formalin or zinc (BD Pharmingen). Fixed tissues were embedded in paraffin, and 3-µm sections were used for all analyses. Histological and immunohistochemical analyses were performed as described (Dutta et al., 2016). For immunohistochemistry on formalin-fixed tissues, sections underwent antigen retrieval by boiling in citrate-acid-based antigen unmasking solution (Vector Labs) for 16 min, while this step was omitted for zinc-fixed tissues. Detection of mouse Nkx3.1 in immunofluorescence assays was enhanced using tyramide amplification (Perkin Elmer) with a horseradish peroxidase (HRP)-conjugated secondary antibody (Invitrogen), followed by incubation with tyramide 488 as described (Dutta et al., 2016). All other antibodies were detected using fluorochrome-coupled secondary antibodies. Details of all antibodies and fixation used for immunohistochemical analyses are provided in Table S4.

Histopathological scoring was performed according to the classification of Park and colleagues (Park et al., 2002). Images were captured using an Olympus VS120 whole-slide scanning microscope. For lineage-tracing experiments, tissues were stained by immunofluorescence and images captured using a Leica TCS SP5 confocal microscope. Quantification of lineage tracing was performed blinded (i.e. without knowledge of the genotype or treatment) by manual counting of cells using 5-10 images from 3-4 independent mice as described (Dutta et al., 2016).

### Flow cytometry analyses of immune cells

Single-cell suspensions from prostate tissue were prepared using the Mouse Tumor Dissociation Kit according to the manufacturer's recommendations (Miltenyi). Cells were Fc-blocked with purified rat anti-mouse CD16/CD32 (clone: 2.4 G2, Becton Dickinson BD) for 30 min at 4°C. Dead cells were discriminated using the LIVE/DEAD (LD) Fixable Near-IR Dead Cell Stain Kit (Thermo Fisher Scientific) and samples were stained for the extracellular and intracellular markers as detailed in Table S4. Staining was visualized by fluorescence-activated cell sorting (FACS) analysis using an LSRFortessa™ (BD Biosciences) and analyzed using FlowJo^®^ (FlowJo LLC). Macrophages are referred to as CD45^+^/B220^−^/CD11b^+^/F4/80^+^, T lymphocytes as CD45^+^/CD11b^−^/CD3^+^, CD4+ T cells as CD45^+^/CD11b^−^/CD3^+^/CD4^+^, CD8+ T cells as CD45^+^/CD11b^−^/CD3^+^/CD8^+^, B cells as CD45^+^/B220^+^/CD11b^−^, and G-MDSCs represent granulocytic myeloid-derived suppressor cells that include neutrophils and are defined as CD45^+^/B220^−^/CD11b^+^/Gr1^high^.

### Bacteria-induced prostatitis model

The CP1 *E. coli* bacterial strain was kindly provided by A. Schaeffer and colleagues (Johns Hopkins University) and was initially isolated from the prostatic secretions of a patient with chronic prostatitis (Rudick et al., 2011). Bacterial cultures and transurethral inoculation were performed as previously described (Simons et al., 2015). Mice were inoculated with a single dose of CP1, or PBS, by transurethral infection.

### Human patient samples

All studies using human patient tissues were performed following protocols approved by the institutional review board of Columbia University Medical Center. Anonymized patient specimens (*n*=11) were obtained from the Molecular Pathology Core, representing patients with clinically-localized prostate cancer (Gleason grade ranging from 6 to 9) who had undergone prostatectomy in the Department of Urology at Columbia University Medical Center. Immunohistochemistry was performed on paraffin-embedded tissues, following standard procedures using a rabbit polyclonal anti-human NKX3.1 antibody and a mouse monoclonal anti E-cadherin antibody (see Table S4 for antibody details). Analyses of expression of specific genes in human prostate cancer was performed using a cohort of primary tumors from TCGA (Cancer Genome Atlas Research, 2015), which includes RNA sequencing data from 290 patients with Gleason 6 and 7 primary prostate tumors. Median of expression was used as a threshold to separate patients in two groups with either a low or high level of *NKX3.1* gene expression (*n*=145 patients per group).

### Statistical analyses

Statistical analyses were conducted using a 2-sample 2-tailed Welch's *t*-test, χ^2^ test or Fisher's exact test as appropriate. Differential gene expression was estimated with a 2-sample 2-tailed Welch’s *t*-test. A *P*-value <0.05 was considered significant. GraphPad Prism software (version 5.0) was used for statistical analyses and data representation.

## Supplementary Material

Supplementary information

First Person interview
