# Supplementary material for: Cooperation of loss of NKX3.1 and inflammation in prostate cancer initiation
Source: Dis Model Mech. 2018 Nov 16;11(11):dmm035139. doi: 10.1242/dmm.035139 (PMC6262819; doi:10.1242/dmm.035139)
Supplement: Supplementary information [file dmm-11-035139-s1.pdf]

FIRST PERSON

First person – Clémentine Le Magnen

First Person is a series of interviews with the first authors of a selection of papers published in Disease Models & Mechanisms, helping early-career researchers promote themselves alongside their papers. Clémentine Le Magnen is first author on 'Cooperation of loss of *NKX3.1* and inflammation in prostate cancer initiation', published in DMM. Clémentine is currently based at the University Hospital of Basel, Switzerland, and was formerly an Associate Research Scientist in the lab of Cory Abate-Shen at Herbert Irving Comprehensive Cancer Center, Columbia University Irving Medical Center, New York, USA. Her research interests include elucidating the mechanisms governing prostate cancer initiation and progression.

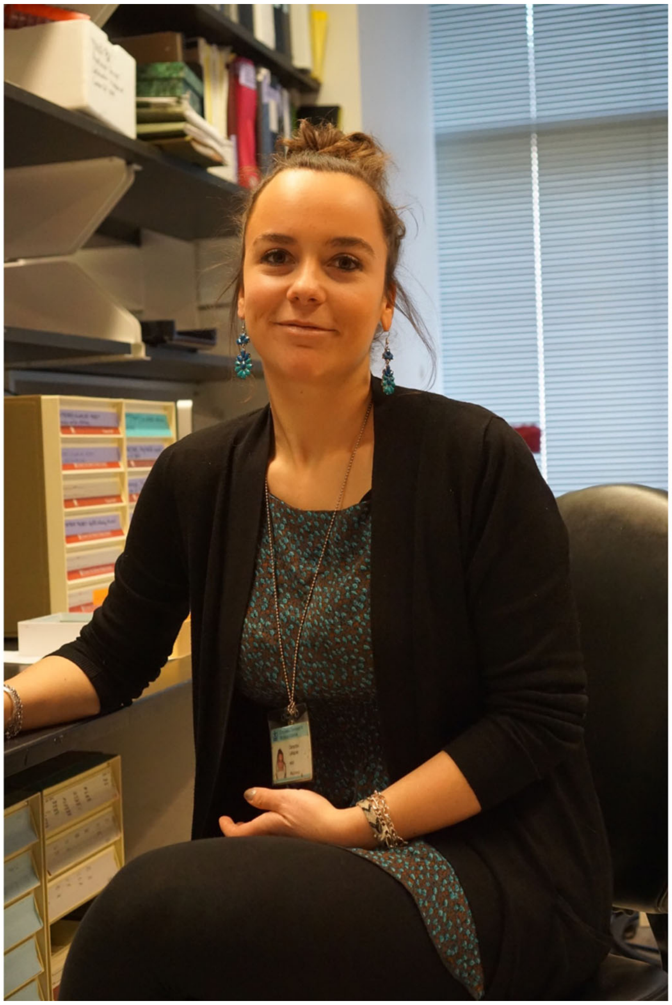

Clémentine Le Magnen

How would you explain the main findings of your paper to non-scientific family and friends?

Prostate cancer is one of the most prevalent cancers and remains a leading cause of cancer-related deaths in men in the United States. As it is clear that prostate cancer is more treatable at early stages, our laboratory has a particular interest in studying how prostate cancer disease arises and grows. Factors contributing to the disease are multiple, and can be genetic and/or environmental. One of the proposed factors is inflammation, which is a normal response to injury or infection that helps damaged tissue to heal. In some cases, however, prolonged inflammation (so-called 'chronic') may damage our healthy cells and this process has been suggested to promote cancer. In this particular study, we used mouse models that are genetically predisposed to develop pre-cancer (i.e. a condition that may develop into cancer but does not necessarily do so). We have observed that these pre-cancerous mouse models are associated with an increase of immune and inflammatory cells at the baseline level. To understand the effect of inflammation, we induced a chronic inflammatory reaction, specifically in their prostate. Under these conditions, they show a higher tendency to develop pre-cancer and their cells display more abnormal characteristics. Taken together with others, these observations suggest that prolonged inflammation may help prostate cancer to grow, especially in the cases where it is combined with specific genetic alterations.

"[…] the implication of our findings is that *NKX3.1* status, combined with markers of inflammation and differentiation, may provide significant insight into the prognosis of men with early-stage prostate cancer."

What are the potential implications of these results for your field of research?

Several groups, including ours, have investigated the contribution of *NKX3.1* loss and that of inflammation to prostate cancer. In this study, using mouse and human data, we report that this relationship is bi-directional and that both factors cooperate to promote early stages of prostate cancer. We also link these findings to processes of aging and altered cellular differentiation. We anticipate that our data will set the basis for future analyses aiming at deciphering the role of specific immune and stromal cell populations in prostate cell differentiation and prostate cancer initiation. At a more translational level, the implication of our findings is that *NKX3.1* status, combined with markers of inflammation and differentiation, may provide significant insight into the prognosis of men with early-stage prostate cancer. Thus, analyses of these factors may enhance models for risk assessment of prostate cancer in a precision prevention context.

What are the main advantages and drawbacks of the model system you have used as it relates to the disease you are investigating?

One main limitation of our model systems is that they are mouse-based and therefore may not fully recapitulate the pathophysiology of the human disease. In particular, the microenvironment and

Clémentine Le Magnen's contact details: Institute of Pathology and Department of Urology, University Hospital of Basel, Basel, Switzerland.
E-mail: Clementine.LeMagnen@usb.ch

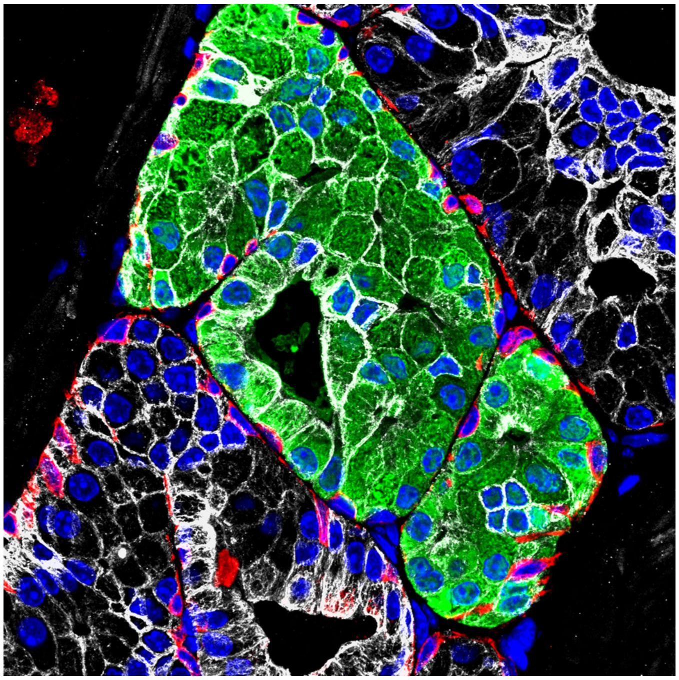

Expression of CK5 (red), CK8 (gray), YFP (green) and DAPI (blue) in the prostate of *CK5-CreER*^{T2}; *Nkx3.1*$^{-/-}$ mice 3 months after CP1 infection.

immune system of murine models may be significantly different from that of humans. For this reason, we have complemented analyses of our mouse models with correlative data from human prostate cancer patients. The significance of our findings for human prostate cancer is also enhanced through the use of an inflammation model that is highly relevant to human prostate cancer. Specifically, we have used the CP1 bacteria model, which was initially isolated from the prostate of a prostatitis patient and is therefore likely to represent a more clinically relevant model of prostatic inflammation, as compared to other models. The other advantage of this model is that it induces long-term chronic inflammation, similar to that observed in the prostate of aged men.

Describe what you think is the most significant challenge impacting your research at this time and how will this be addressed over the next 10 years?

I think that one of the most significant challenges impacting prostate cancer research has been the lack of clinically relevant human models to study the disease. Fortunately, recent technical advances have enabled the establishment of patient-derived 3D culture models, such as organoids, and *in vivo* models, such as xenografts. While these models represent promising tools, they do not recapitulate the native tissue microenvironment and are usually characterized by a limited take rate. Thus, much work has to be done to make these systems more efficient and relevant, and to better model tumor heterogeneity, which is also a significant challenge in the cancer research field.

"[…] too many young scientists give up their scientific career because they believe that they did not choose the right path and have not been well mentored/advised by more experienced scientists."

What changes do you think could improve the professional lives of early-career scientists?

In my opinion, too many young scientists give up their scientific career because they believe that they did not choose the right path and have not been well mentored/advised by more experienced scientists. One way to improve this matter may be to better inform and mentor future scientists early in their career. Specifically, they should be informed about the different career opportunities in science, the paths towards the achievement of their career goal, and also their requirements, such as mobility and specific skills. This may be implanted through special career information and mentoring sessions at the university, that would ideally involve the participation of committed early and advanced scientists (with an academic and industry background). In addition, as funding is a crucial issue for early career scientists, we should aim at establishing more funding schemes that are specifically dedicated to them.

What's next for you?

After five years of postdoctoral training at Columbia Medical Center, I recently moved to a new position at the University Hospital of Basel in Switzerland. There, I am working at establishing my own research team that will focus on prostate cancer translational research and, in particular, identifying molecular and cellular determinants of prostate cancer progression.

Reference

Le Magnen, C., Virk, R. K., Dutta, A., Kim, J. Y., Panja, S., Lopez-Bujanda, Z. A., Califano, A., Drake, C. G., Mitrofanova, A. and Abate-Shen, C. (2018). Cooperation of loss of *NKX3.1* and inflammation in prostate cancer initiation. *Dis. Model. Mech.* **11**, dmm035139.
